# Arsenic Reduction in Drinking Water and Improvement in Skin Lesions: A Follow-Up Study in Bangladesh

**DOI:** 10.1289/ehp.1205381

**Published:** 2012-10-10

**Authors:** Wei Jie Seow, Wen-Chi Pan, Molly L. Kile, Andrea A. Baccarelli, Quazi Quamruzzaman, Mahmuder Rahman, Golam Mahiuddin, Golam Mostofa, Xihong Lin, David C. Christiani

**Affiliations:** 1 Department of Environmental Health, Harvard School of Public Health, Boston, Massachusetts, USA; 2 College of Public Health and Human Sciences, Oregon State University, Corvallis, Oregon, USA; 3 Dhaka Community Hospital, Dhaka, Bangladesh; 4 Department of Biostatistics, Harvard School of Public Health, Boston, Massachusetts, USA

**Keywords:** arsenic, Bangladesh, change, recovery, skin lesion

## Abstract

Background: Chronic exposure to arsenic is associated with skin lesions. However, it is not known whether reducing arsenic exposure will improve skin lesions.

Objective: We evaluated the association between reduced arsenic exposures and skin lesion recovery over time.

Methods: A follow-up study of 550 individuals was conducted in 2009–2011 on a baseline population of skin lesion cases (*n* = 900) previously enrolled in Bangladesh in 2001–2003. Arsenic in drinking water and toenails, and skin lesion status and severity were ascertained at baseline and follow-up. We used logistic regression and generalized estimating equation (GEE) models to evaluate the association between log_10_-transformed arsenic exposure and skin lesion persistence and severity.

Results: During the study period, water arsenic concentrations decreased in this population by 41% overall, and 65 individuals who had skin lesions at baseline had no identifiable lesions at follow-up. In the adjusted models, every log_10_ decrease in water arsenic and toenail arsenic was associated with 22% [odds ratio (OR) = 1.22; 95% CI: 0.85, 1.78] and 4.5 times (OR = 4.49; 95% CI: 1.94, 11.1) relative increase in skin lesion recovery, respectively. In addition, lower baseline arsenic levels were significantly associated with increased odds of recovery. A log_10_ decrease in toenail arsenic from baseline to follow-up was also significantly associated with reduced skin lesion severity in cases over time (mean score change of –5.22 units; 95% CI: –8.61, –1.82).

Conclusions: Reducing arsenic exposure increased the odds that an individual with skin lesions would recover or show less severe lesions within 10 years. Reducing arsenic exposure must remain a public health priority in Bangladesh and in other regions affected by arsenic-contaminated water.

Globally, millions of people are exposed to arsenic from drinking contaminated water (Brammer and Ravenscroft 2009). Inorganic arsenic is classified as a Group 1 human carcinogen (International Agency for Research on Cancer 2004), and chronic exposure to arsenic in drinking water is associated with increased risk of skin, bladder, lung, and kidney cancer (Fernandez et al. 2012; Guo et al. 2001; Hopenhayn-Rich et al. 1998; Wu et al. 1989). Chronic exposure to arsenic is also associated with increased risk of skin lesions, cardiovascular diseases, lung function, hypertension, and reproductive and neurological disorders (Chattopadhyay et al. 2010; Chen et al. 2009; Milton et al. 2005; Rahman 2002).

Arsenic-contaminated groundwater in Bangladesh is a public health concern due to the use of shallow tube wells as part of a public health campaign to reduce the burden of waterborne diseases (Bagla and Kaiser 1996; Chakraborty and Saha 1987). It has been estimated that 45% of the population in Bangladesh was exposed to arsenic concentrations greater than the World Health Organization (WHO) recommended limit of 10 μg/L, and 28% were exposed to concentrations greater than the Bangladesh standard of 50 μg/L (Kinniburgh and Smedley 2001). Efforts to remediate arsenic-contaminated water in Bangladesh are ongoing and include testing tube wells, labeling unsafe wells, and installing new arsenic-free water sources and point-of-use filters (Joya et al. 2006; UNICEF 2008).

Skin lesions are the first visible symptom of chronic arsenic exposure (Chakraborty and Saha 1987; Rahman et al. 2001; Tseng et al. 1968) and are considered to be precursors of arsenic-induced cancers. These skin lesions are highly associated with skin cancers and other malignancies, and they are highly associated with arsenical skin cancers (Cuzick et al. 1982, 1984, 1992; Tseng 1977). Prospective cohort studies conducted in Bangladesh have provided good evidence that the incidence of skin lesions increases with arsenic levels over time (Argos et al. 2011), but few studies have examined whether skin lesions improve if arsenic exposures are reduced. In addition, most previous studies have evaluated the association between arsenic exposure and skin lesions as a dichotomous outcome only, without also considering the severity of skin lesions.

In 2009 we followed up individuals who participated in a case–control study of environmental and genetic risk factors for arsenic-related skin lesions in 2001–2003. Our main objective was to determine whether arsenic exposures decreased over time, and whether arsenic reduction was associated with a reduction in skin lesions. We estimated associations between changes in arsenic exposure and the prevalence and severity of skin lesions at follow-up among participants who had skin lesions at baseline.

## Methods

*Study population*. In 2001–2003 (baseline), we enrolled 900 individuals who were diagnosed with arsenic-related skin lesions and 900 age- and sex-matched controls in Pabna, Bangladesh, into a case–control study to identify factors that influence susceptibility to arsenic-related skin lesions, as previously described by Breton et al. (2006). In a follow-up study during 2009–2011, 845 (93.9%) of the original 900 participants with skin lesions were successfully recontacted, and 550 cases (61.1% of the 900) agreed to participate in the follow-up study. Of those contacted, the main reasons for nonparticipation were refusal (53%), moved away from the district (38%), and mortality (9%).

All individuals in the baseline case–control study participated in Dhaka Community Hospital’s arsenic awareness program, which provided information on the health effects of arsenic exposure, actions that individuals could take to reduce their exposure, and the importance of a diet rich in fruits and vegetables. Dhaka Community Hospital and their affiliated clinics also worked with impacted villages to install arsenic-free water sources through shallow dug wells, larger Indira wells, filtered surface water, and rainwater harvesting (Joya et al. 2006; Quamruzzaman et al. 2001). Efforts to provide new water sources were targeted for households that were considered highly exposed (> 50 μg/L).

At the time of follow-up, all participants underwent a physical examination, and skin lesion status was reassessed by the same physician who conducted the baseline exam in 2001–2003 and who was blinded to the arsenic concentration in the participants’ drinking water. The follow-up study protocol was approved by the institutional review boards of the Harvard School of Public Health and Dhaka Community Hospital. Informed consent was provided by every participant prior to participation in the follow-up study.

*Questionnaires and interviews*. Trained interviewers administered questionnaires to collect sociodemographic information, drinking water history, medical history, lifestyle factors, dietary information, water consumption (liters of water/liquid ingested per day), and residential history including identification of the primary water source (tube well), years of use, and use of a previous tube well. Interviewers in the follow-up study were blinded to the participants’ disease status and arsenic exposure at baseline.

*Exposure assessment.* We collected a water sample from each participant’s primary drinking source; 45 mL water was placed in a 50-mL Falcon tube, and one drop (0.1 mL) of pure trace metal grade nitric acid was added to preserve the samples for trace metal analysis. Samples were stored at room temperature prior to analysis. Arsenic concentration was analyzed in each sample by inductively coupled plasma mass spectrometry (ICP-MS) following U.S. Environmental Protection Agency Method 200.8 (U.S. EPA 1994). Analyses were performed by the same laboratory that conducted the baseline measurements (Environmental Laboratory Services, North Syracuse, New York). For quality control, instrument performance was validated using repeated measurements of standard reference water (PlasmaCAL Multi-Element QC Standard 1; SCP Science, Canada) with an average percent recovery of 95%. Ten percent of the samples were randomly selected and analyzed in duplicate to confirm reliability. The average percent difference between duplicates was 2.5%. The limit of detection (LOD) of arsenic was 1 μg/L; samples with concentrations < LOD were assigned a value of 0.5 μg/L.

Nail clippings were collected from each participant, placed in an envelope, and stored at room temperature in a dry location. External contamination was removed by sonicating the nails with 1% Triton X-100 solution for 20 min (Sigma-Aldrich Inc., St. Louis, MO, USA). Samples were digested at room temperature for at least 36 hr following the same protocols as used in the baseline case–control study (Amarasiriwardena et al. 1998). Total arsenic was measured using ICP-MS (Model 6100 DRC; PerkinElmer, Norwalk, CT, USA), and each sample was subjected to five replicate analyses. The average LOD was 0.02 μg/g; no samples had arsenic concentrations < LOD. Instrument performance and the digestion process were validated using standard reference material (SRM) 1643d and SRM 1643e (Trace Elements in Water; National Institute of Standards and Technology, Gaithersburg, MD, USA). The average percent recovery of SRMs was 86.5%, and the average percent hair SRM across batches was 73.6%.

*Outcome assessment.* Two different metrics for skin lesions were assessed: a dichotomous skin lesion status (yes/no) and a continuous skin lesion severity score.

Dichotomous physician-diagnosed skin lesions. *“*Melanosis” (yes/no) was defined as any diffuse or spotted lesion characterized by dark pigmentation on the face, oral cavity, neck, upper and lower limbs, chest, or back. “Keratosis” (yes/no) was defined as any diffuse or spotted lesion characterized by hard and roughened skin elevations observed on the palm or dorsum of the hands and/or the sole or plantar of the foot. “Hyperkeratosis” (yes/no) was defined as extensively thickened keratosis observed on the palm or dorsum of the hands and/or the sole or plantar of the foot that are easily visible from a distance. “Leukomelanosis” (yes/no) was defined as depigmentation characterized by black and white spots present anywhere on the body. At follow-up, “persistent cases” were defined as participants who had at least one type of arsenic-induced skin lesion (melanosis, keratosis, hyperkeratosis, or leukomelanosis) at baseline and at follow-up. At the time of the follow-up physical examination, 65 (11.8%) individuals who had skin lesions at baseline had no visible skin lesions and were classified as “recovered cases.”

Continuous skin lesion severity score. To assess the severity of skin lesions, the study physician noted the presence of any skin lesion and assigned a score of 0, 1, or 2 (indicating no lesion, a mild lesion, or a severe lesion, respectively) in *a*) each one of 11 specified anatomical regions for both diffuse and spotted melanosis (face, oral cavity, neck, arm, dorsum, palm, chest, back, leg, plantar and sole; maximum possible score of 22 + 22 = 44); *b*) each one of 4 specified anatomical regions for both diffuse and spotted keratosis (palm, dorsum, sole, and plantar; maximum possible score of 8 + 8 = 16); and *c*) each one of 4 specified anatomical regions for hyperkeratosis (palm, dorsum, sole, and plantar; maximum possible score of 8); and also noted the presence or absence of leukomelanosis anywhere on the body (maximum score of 1). The physician then summed the individual scores across the anatomical regions to create a continuous severity score that ranged from 0 to 69. This overall severity score takes into account the possibility for an individual to have more than one type of skin lesion, each of different severity. This approach was adapted from a method used by Ahsan et al. (2006) to quantify arsenic-induced skin lesions, which was based on methodology originally used to determine the extent of body involvement in burn patients. Color photographs of representative mild (severity score = 1) and severe (severity score = 2) skin lesions are shown in Supplemental Material, Figure S1 (http://dx.doi.org/10.1289/ehp.1205381). Photographs of lesions were taken for all the skin lesion cases at baseline. A random sample (5%) of persistent cases were independently scored by a dermatologist to evaluate inter-rater reliability, which indicated mean consistency of 72% for the overall scores for each participant in the subsample with previous diagnosis.

*Statistical analysis.* Our analysis was limited to individuals with skin lesions diagnosed at baseline in 2001–2003 who had follow-up information in 2009–2011. All covariates were determined at both baseline and follow-up. Covariates such as age, sex, smoking status, and betel nut chewing were compared between followed-up and nonparticipating cases, as well as persistent cases and recovered cases at follow-up, using Fisher’s exact test for categorical variables, Welch’s *t*-test for normally distributed variables, and Wilcoxon rank-sum test with continuity correction for non-normally distributed continuous variables. All arsenic variables were log_10_-transformed because arsenic was right-skewed. Arsenic change for participant *i* (ΔAs*_i_* = log_10_As*_i_*_0_ – log_10_As*_i_*_1_) was defined as the reduction of arsenic levels between follow-up (As*_i_*_1_) and baseline arsenic levels (As*_i_*_0_).

First, we evaluated the association between reduction in arsenic levels and skin lesion recovery (*n* = 65 recovered cases vs. 485 persistent cases) using logistic regression models:

logit(π*_i_*) = β_0_ + β_1_log_10_As*_i_*_0_ + β_2_ΔAs*_i_* + α^T^Z*_i_*, [1]

where π*_i_* is the probability of full recovery status of subject *i* at follow-up; β_0_ is the intercept; β_1_ is the log odds of skin lesion recovery associated with a log_10_ unit increase in As*_i_*_0_; β_2_ is the log odds of skin lesion recovery associated with a log_10_ unit decrease in ΔAs*_i_*; α^T^ is a row vector of regression coefficient estimates for covariates at follow-up (T denotes vector transpose); and Z is a vector of covariates at follow-up. Covariates included in the final model were selected *a priori*, including age (continuous), sex, education (≤ primary, secondary–college, ≥ graduate), body mass index (BMI; continuous), smoking status (never vs. ever or current smoker), and chewing betel nuts (yes or no) at follow-up.

Second, we used linear regression with generalized estimating equations (GEE) to estimate the association between changes in skin lesion severity between baseline and follow-up among all cases in relation to changes in biomarkers of arsenic exposure (water, toenails) over time, controlling for baseline arsenic levels (Diggle et al. 2002). The GEE analysis accounts for within-subject correlation between baseline and follow-up severity scores. To improve analysis power, we used all of the observed data from the 900 subjects, including those subjects who have both baseline and follow-up data (*n* = 550) and those who only had the baseline data (*n* = 350). The model used in the GEE analysis can be written as

E(Score*_it_*) = β_0_ + β_1_log_10_As*_i_*_0_ + β_2_*t_i_* + β_3_ΔAs*_it_* + β_4_log_10_As*_i_*_0_*t_i_ + α^T^Z*_i_*, [2]

where Score*_it_* is severity score of subject *i* at time *t* (0 = baseline, 1 = follow-up); ΔAs*_it_* is the change in arsenic level in subject *i* at time *t* with ΔAs*_i_*_0_ = 0. At baseline (*t* = 0) the model simplifies to E(Score_i0_) = β_0_ + β_1_log_10_As_i0_ + α^T^Z_i_ and at follow-up (*t* = 1) the model simplifies to E(Score_i1_) = β_0_ + β_1_log_10_As_i0_ + β_2_ + β_3_ΔAs_i1_ + β_4_log_10_As_i0_ + α^T^Z_i_ such that the change in the severity score between baseline and follow-up for individual *i* (ΔScore*_i_*) is represented by E(Score*_i_*_1_)–E(Score*_i_*_0_), that is,

E(ΔScore*_i_*) = β_2_ + β_3_ΔAs*_i_* + β_4_log_10_As*_i_*_0_. [3]

Hence, β_1_ is the change in mean baseline severity score for every log_10_ unit increase in baseline arsenic levels; β_3_ is the change in ΔScore for every log_10_ unit decrease in ΔAs*_i_* (i.e., the estimated effect of the change in arsenic levels over time on the change in the severity score over time); and β_4_ is the change in ΔScore for every log_10_ unit increase in baseline arsenic levels (i.e., the estimated effect of baseline arsenic levels on the change in the severity score over time).

Table 1 shows that the subjects who dropped out of the study were younger and had a lower BMI and higher arsenic level at baseline. To account for potential bias due to dropout of these cases at follow-up, we performed an inverse probability weighted (IPW) GEE model by assigning weights according to each individual’s estimated probability of participating in the follow-up study using logistic regression on age, BMI, and arsenic exposures at baseline and found virtually unchanged results as the unweighted GEE model [see Supplemental Material, Table S1 (http://dx.doi.org/10.1289/ehp.1205381)]. All statistical analyses were conducted using R, version 2.13.1 (http://www.R-project.org/), and SAS, version 9.2 (SAS Institute Inc., Cary, NC, USA). All tests were conducted as two-sided, and *p* < 0.05 was considered significant.

**Table 1:** Characteristics of follow-up cases (*n* = 550) and nonparticipating cases (*n* = 350) recruited in Pabna, Bangladesh in 2001–2003.

Characteristic	Follow-up cases		Nonparticipating casesa	
	Baseline	Follow-up	Baseline	p-Valueb
Sex								
Male	340 (61.8)	340 (61.8)	216 (61.7)	0.96
Female	210 (38.2) )	210 (38.2)	134 (38.3)
Age (years)	34.2 ± 11.8	41.4 ± 12.5	32.5 ± 12.8	0.05
Education								
None–primary school	351 (63.9)	408 (74.2)	216 (61.7)	0.15
Secondary school–college	180 (32.8)	111 (20.2)	113 (32.3)
≥ Graduate school	18 (3.3)	31 (5.6)	21 (6)
BMI (kg/m2)	20.2 ± 3.12	20.6 ± 3.10	19.7 ± 2.93	0.01
Smoking status (only in males)								
Ever	158 (46.6)	168 (49.4)	96 (45.3)	0.79
Never	181 (53.4)	172 (50.6)	116 (54.7)
Chew betel nuts								
Yes	162 (29.6)	170 (30.9)	94 (27.0)	0.46
No	386 (70.4)	380 (69.1)	254 (73.0)
Water arsenic (µg/L)	212.9 ± 301.9	125 ± 227	263.2 ± 314.3	0.002
Toenail arsenic (µg/g)	6.18 ± 7.95	6.0 ± 8.15	7.33 ± 8.53	0.009
Severity score	16.5 ± 14.8	34.4 ± 15.6	16.5 ± 15.4	0.67
Data shown are mean ± SD for continuous variables and n (%) for categorical variables. aReasons for nonparticipation: 53% refused to participate in follow-up study, 38% had moved away, and 9% had died. bp‑Values comparing baseline characteristics between follow-up and nonparticipating subjects obtained by Welch’s t-test for normally distributed continuous variables, by Wilcoxon rank sum test with continuity correction for nonnormally distributed continuous variables, and by Fisher’s exact test for categorical variables.								

## Results

In total, 61.1% of the 900 cases (*n* = 550) from the original case–control study participated in the follow-up. At baseline (2001–2003), cases who participated in the follow-up study had higher BMI, used tube wells that had lower arsenic concentrations, and had lower toenail arsenic than cases who did not participate in follow-up (*p* < 0.05) (Table 1).

**Figure 1:**
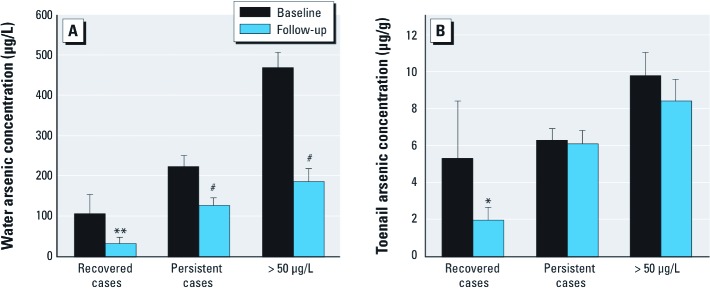
Reduction of mean arsenic concentrations in water (*A*) and toenail (*B*) between baseline (2001–2003) and follow-up (2009–2011) in the recovered cases (*n *= 65), persistent cases (*n *= 485), and subjects exposed to > 50 µg/L of baseline water arsenic (*n *= 237). Error bars represent 1.96 × SE. *p < 0.05, **p < 0.01, and ^#^*p*< 0.001, by Welch’s *t*-test

Sixty-five participants had no skin lesions identified at follow-up (recovered cases), whereas 485 participants still had skin lesions (persistent cases) (Table 2). At follow-up, the recovered cases were younger (mean, 39.2 years compared with 41.8 years; *p* = 0.02), had higher average BMI (21.0 compared with 20.0; *p* = 0.01), and did not chew betel nuts as frequently (18.5% versus 32.6%; *p* = 0.02) than persistent cases. Overall, drinking water arsenic concentrations declined significantly in the follow-up study population (125 ± 227 μg/L compared with 213 ± 302 μg/L at baseline; *p* < 0.001) (Table 1, Figure 1). Among the recovered cases, drinking water arsenic concentrations declined, on average, by 70.5%, from a mean of 105.4 µg/L in 2001 to 31.1 µg/L in 2009; however, in persistent cases there was a mean reduction of only 43.4%, from 221.6 µg/L in 2001 to 125.4 µg/L in 2009, even though the absolute change in water arsenic concentrations was greater for the persistent cases than recovered cases (Table 2).

**Table 2:** Characteristics of persistent cases (*n* = 485) and recovered cases (*n* = 65) at follow-up in Pabna, Bangladesh, 2009–2011.

Characteristic	Persistent cases	Recovered cases	p-Valueb
Sex						
Males	294 (60.6)	44 (67.7)	0.13
Females	191 (39.5)	21 (32.3)
Age (years)	41.8 ± 12.5	39.2 ± 12.8	0.02
Education level
None–primary school	367 (75.7)	41 (63.1)	0.09
Secondary school–college	92 (19.0)	19 (29.2)
≥ Graduate school	26 (5.36)	5 (7.7)
BMI	20.0 ± 3.11	21.0 ± 3.03	0.01
Smoking status (only in males)
Ever	145 (49.3)	23 (50.0)	0.98
Never	149 (50.7)	23 (50.0)
Chew betel nuts
Yes	158 (32.6)	12 (18.5)	0.02
No	327 (67.4)	53 (81.5)
Baseline water arsenic (µg/L)	222 ± 309	105 ± 196	0.004
Follow-up water arsenic (µg/L)	125 ± 229	31.1 ± 64.6	0.002
Change in water arsenic (µg/L)	–97.0 ± 331	–47.4 ± 155	0.51
Baseline water arsenic ≥ 50 µg/L
Yes	216 (44.5)	21 (32.3)	0.06
No	269 (55.5)	44 (67.7)
Follow-up water arsenic ≥ 50 µg/L
Yes	159 (32.8)	9 (13.8)	0.001
No	326 (67.2)	56 (86.2)
Baseline toenail arsenic (µg/g)	6.29 ± 7.09	5.31 ± 12.7	0.002
Follow-up toenail arsenic (µg/g)	6.07 ± 8.23	1.95 ± 2.79	< 0.001
Change in toenail arsenic (µg/g)	–0.43 ± 7.40	–3.14 ± 12.7	0.13
Baseline severity score	16.0 ± 14.6	8.14 ± 11.7	< 0.001
Follow-up severity score	39.0 ± 9.75	0	—
Data are shown as mean ± SD for continuous variables or n (%) for categorical variables. p-Values comparing persistent and recovered cases were obtained using Welch’s t-test for normally distributed continuous variables and Wilcoxon rank sum test with continuity correction for non-normally distributed continuous variables; Fisher’s exact test was used for categorical variables.						

Participants exposed at baseline to water arsenic > 50 µg/L (the Bangladesh recommended maximum contaminant level of arsenic) were targeted for mitigation activities; of these, 40.4% had water arsenic levels < 50 µg/L at follow-up (Table 2). A significantly lower proportion of recovered cases were exposed to ≥ 50 µg/L water arsenic at follow-up compared with persistent cases (13.8% versus 32.8%). Toenail arsenic levels were significantly reduced from baseline to follow-up in the recovered cases (5.31 µg/g versus 1.95 µg/g) but not in the persistent cases (6.29 µg/g vs. 6.07 µg/g), which supports our hypothesis that reduction in arsenic exposure is the main driving force for their recovery. Among persistent cases, water arsenic levels also decreased over time by > 200% (mean decrease of 97 µg/L), yet mean severity of skin lesions in this group increased from 16 units in 2001 to 39 units in 2009 (*p* < 0.05) (Table 2). This increase in skin lesion severity may be due to continued arsenic exposure because the average drinking water arsenic concentration in this group remained at 125 µg/L, and as noted above, there was little change in average toenail arsenic concentrations in persistent cases.

The association between reduction in arsenic level and skin lesion recovery (yes/no) in cases at follow-up was assessed using logistic regression (Table 3). For every log_10_ unit decrease in water arsenic, there was a 1.22 times increase in odds of skin lesion recovery [odds ratio (OR) = 1.22; 95% confidence interval (CI): 0.85, 1.78], and for every log_10_ unit decrease in toenail arsenic, there was a significant 4.49 times increase in odds of skin lesion recovery (OR = 4.49; 95% CI: 1.94, 11.1), after adjusting for age, sex, education, smoking status, betel nut chewing, BMI, and baseline arsenic. In other words, the greater the reductions in arsenic levels in both water and toenail, the higher the probability of recovering from skin lesions over time. We also observed significant associations with baseline arsenic levels. For every log_10_ unit increase in baseline water arsenic, there was a 41% relative decrease in odds of skin lesion recovery (OR = 0.59; 95% CI: 0.41, 0.81), and for every log_10_ unit increase in baseline toenail arsenic, there was an 80% relative decrease in odds of skin lesion recovery (OR = 0.20; 95% CI: 0.08, 0.44), after adjusting for all other covariates.

**Table 3:** Change in odds of skin lesion recovery at follow-up examination (*n* = 65) for every log_10_ unit decrease in arsenic concentration between baseline (2001–2003) and follow-up (2009–2011) among baseline cases (*n* = 541) who had follow-up data.

	Exposure (log10)	Crude		Adjusteda	
		Change in odds (95% CI)	p-Value	Change in odds (95% CI)	p-Value
Decreaseb	Water arsenic	1.37 (0.98, 1.96)	0.072	1.22 (0.85 1.78)	0.28
	Toenail arsenic	5.32 (2.38, 12.6)	< 0.001	4.49 (1.94, 11.1)	< 0.001
Baselinec	Water arsenic	0.56 (0.40, 0.77)	< 0.001	0.59 (0.41, 0.81)	0.002
	Toenail arsenic	0.18 (0.08, 0.39)	< 0.001	0.20 (0.08, 0.44)	< 0.001
aAdjusted for age, sex, smoking status, betel nut chewing, education, BMI, and baseline arsenic. bDecrease between baseline and follow-up. cFor every log10 unit increase in baseline arsenic level.										

We used linear regression with GEEs to assess the association between longitudinal arsenic level profiles and skin lesion severity profiles while accounting for possible correlation among repeated measures over time within the same subject. For every log_10_ unit reduction in water arsenic, the mean skin lesion severity score was reduced by 0.70 units (95% CI: –2.18, 0.78), and for every log_10_ unit reduction in toenail arsenic, the mean skin lesion severity score was significantly reduced by 5.22 units (95% CI: –8.61, –1.82), after adjusting for age, sex, education, smoking status, betel nut chewing, BMI, and baseline arsenic (Table 4). For every log_10_ unit increase in baseline water arsenic, the mean skin lesion severity score was reduced by 0.87 units (95% CI: –2.85, 0.18), after adjusting for all other covariates.

**Table 4:** Decrease in mean severity score associated with a log_10_ unit decrease in arsenic concentration in cases (*N* = 550) at the follow-up examination (2009–2011) using linear regression fitted using generalized estimating equation (GEE).

	Exposure (log10)	Crude		Adjusteda	
		Mean score change (95% CI)	p-Value	Mean score change (95% CI)	p-Value
Decreaseb	Water arsenic	–0.84 (–2.34, 0.65)	0.27	–0.70 (–2.18, 0.78)	0.35
	Toenail arsenic	–5.74 (–9.15, –2.33)	< 0.001	–5.22 (–8.61, –1.82)	0.003
Baselinec	Water arsenic	–1.14 (–2.63, 0.36)	0.13	–1.34 (–2.85, 0.18)	0.08
	Toenail arsenic	0.24 (–3.04, 3.52)	0.89	–0.09 (–3.41, 3.22)	0.96
aAdjusted for age, sex, smoking status, betel nut chewing, education, BMI, and baseline arsenic. bDecrease between baseline and follow-up. cFor every log10 unit increase in baseline arsenic level.										

## Discussion

Our results show that arsenic remediation activities and safe water programs have successfully reduced arsenic exposures in this population. Individuals who at baseline were drinking water from a tube well that contained arsenic at concentrations that exceeded the Bangladesh drinking water standard (> 50 μg/L) had the most dramatic decreases in arsenic exposure, indicating that these individuals were able to reduce their exposures and that Dhaka Community Hospital’s arsenic remediation program was effective at helping to encourage and motivate individuals to change their drinking water sources and behaviors that lead to arsenic exposure. We observed a decrease in drinking water arsenic over time that was associated with increased probability that skin lesions would improve within a period of 10 years. These findings highlight the importance and effectiveness of long-term community-based arsenic remediation efforts in Bangladesh.

As expected, we observed that individuals who had lower baseline arsenic exposures had significantly higher odds of recovery at follow-up. Recovered cases were exposed to lower baseline water arsenic levels than persistent cases, and had lower water arsenic levels at follow-up. They also had much greater reduction in toenail arsenic levels compared with persistent cases. These results suggest that reduction of water arsenic over time, together with lower baseline arsenic levels, are strongly associated with recovering from skin lesions. We also observed a borderline significant association between baseline water arsenic and reduced severity of skin lesions, which might be due to the possibility that individuals with higher baseline arsenic were more targeted to change their water sources and therefore have reduced skin lesions severity at follow-up. However, persistent cases that were highly exposed at baseline and reduced their exposure to arsenic-contaminated drinking water may need to further reduce their exposures or may require a longer period of time to fully recover from their skin lesion symptoms.

Very little information is available regarding the effects of reducing arsenic exposure on skin lesion recovery. In an early clinical report that reviewed all prior literature on arsenical cancers, Neubauer (1947) stated that early-stage arsenic-induced lesions can spontaneously disappear when the use of medications containing arsenic is reduced. However, no quantitative population study has yet been published, and the biological mechanisms are still poorly understood. Banerjee et al. (2008) demonstrated that exposed individuals with hyperkeratosis have significantly higher DNA damage (measured by a chromosomal aberration assay in lymphocytes) and less DNA repair capacity (measured using a challenge assay in whole blood) than those without skin lesions. Other studies reported that oxidative DNA damage (urinary 8-hydroxy-2´-deoxyguanosine) was repaired and returned to normal in patients 180 days after accidental oral intake of arsenic trioxide (Mahata et al. 2004; Yamauchi et al. 2004). The reduction of water arsenic was significant in our population, especially in the recovered group. Therefore, repair of DNA damage may explain why we observed reversibility of skin lesions after arsenic exposures decreased.

In our population, the most common arsenic remediation intervention for individuals exposed to > 50 μg/L was switching to a safe well containing lower arsenic concentrations. In a prospective study, Chen et al. (2007) evaluated the effectiveness of this approach by comparing urinary arsenic concentrations at baseline and 2 years later, and found a 46% reduction in mean urinary arsenic concentrations among those who switched wells. Together, these results suggest that switching wells is an acceptable remediation strategy that can reduce an individual’s exposure to arsenic-contaminated drinking water and improve public health outcomes. Besides remediation activities to reduce arsenic exposure from drinking water, numerous studies have also shown dietary supplements to play an important role in influencing arsenic toxicity both in the human population and *in vitro* in blood cultures (Biswas et al. 2010; Gamble et al. 2006, 2007; Heck et al. 2007; Tiwari and Rao 2010). However, a pilot supplementation trial that provided randomized subjects with supplements vitamin E, selenium, and a combination of the two for 6 months did not find a significant mean decrease in skin lesion scores (Verret et al. 2005). Compared with the results from implementing dietary supplementation, we have shown that lowering the arsenic levels in drinking water may result in a more significant improvement of skin lesion severity and eventually recovery.

Some limitations of our study include possible exposure misclassification, misclassification of mild skin lesions, lack of confirmation of recovered cases by an independent physician, and selection bias due to individuals who were not included in the follow-up because of refusal to participate or loss to follow-up. Subjects could have been drinking water from other sources that were not accounted for; because we have only one measurement of water arsenic for each subject in any one period, exposure misclassification is possible. However, we also observed associations between reduced toenail arsenic, a biomarker of internal dose, and the odds of skin lesion recovery. False-positive misclassification of mild skin lesions such as melanosis or leukomelanosis was possible; participants who were actually controls were misclassified as cases at baseline and reevaluated as recovered cases at follow-up. However, the same physician assessed the cases at both baseline and follow-up. Furthermore, the physician was blinded to each participant’s baseline case status and arsenic exposure. Finally, because this was a follow-up of a case–control study with a substantial percentage of participants who were not followed up, there might be potential selection bias if cases that were not followed up had higher arsenic exposures and developed more severe skin lesions due to arsenic toxicity. However, when we compared baseline characteristics of the two groups (followed-up and nonparticipating cases) in Table 1, we found no significant differences in skin lesion severity scores or risk factors for skin lesions, such as smoking and chewing betel nuts.

Strengths of our study include *a*) a relatively long follow-up period of almost 10 years; *b*) individual exposure assessment for arsenic, which included external environmental exposure from drinking water and cumulative internal dose in toenails as biomarkers at both baseline and follow-up; and *c*) assessment of skin lesions on a continuous scale to provide additional information on their severity. Given the nature of the study design, we were able to examine the effects of decreased arsenic levels on skin lesions. Although a few prospective studies are investigating the association between arsenic and skin lesions, we are not aware of published studies concerning associations between reductions in arsenic exposures over time and skin lesion recovery or changes in skin lesion severity over time. The present study is one of the first studies to evaluate the effects of reduced arsenic exposures via drinking water by assessing improvements in skin lesions over time.

Our results strongly show that reduced arsenic exposures not only increased the odds of skin lesion recovery but also reduced the severity of skin lesions among participants with skin lesions over time. Intervention efforts should target individuals with skin lesions to reduce their arsenic levels as early as possible in order to increase their chances of skin lesion recovery. Arsenic-induced skin lesions are often the first visible symptom of arsenic poisoning and are therefore often indicative of more arsenic-related morbidities to follow; thus, reducing arsenic exposures over time holds promises of full recovery from skin lesions as long as effective remediation efforts are put in place.

## Conclusion

We found a substantial reduction in water arsenic levels in our study population, reflecting considerable efforts to reduce arsenic intake from drinking water in this region over the study period. We found that the reduction in arsenic exposure was associated with increasing odds of recovery from skin lesions and with reduced severity of persistent lesions at follow-up. Future studies with extended follow-up are needed to assess whether this reduction in the presence and severity of skin lesion results in reduced risks of cancers and other arsenic-related diseases with longer latency periods.

## Correction

In Table 1 of the manuscript originally published online, the values were incorrect for smoking status at baseline for both follow-up cases and nonparticipating cases. These values have been corrected here.

## Supplemental Material

(8.8 MB) PDF
