# Supplementary material for: Arsenic Reduction in Drinking Water and Improvement in Skin Lesions: A Follow-Up Study in Bangladesh
Source: Environ Health Perspect. 2012 Oct 10;120(12):1733–8. doi: 10.1289/ehp.1205381 (PMC3548283; doi:10.1289/ehp.1205381)
Supplement: (8.8 MB) PDF [file ehp.1205381.s001.pdf]

Supplemental Material

Arsenic Reduction in Drinking Water and Improvement in Skin Lesions: A Follow-Up Study in Bangladesh

Wei Jie Seow, Wen-Chi Pan, Molly L. Kile, Andrea A. Baccarelli, Quazi Quamruzzaman, Mahmuder Rahman, Golam Mahiuddin, Golam Mostofa, Xihong Lin, David C. Christiani*

Table of Contents

1. Figure S1 - Colored photographs of skin lesions showing different types and levels of severity on patient's chest and palm.

2. Table S1 – Comparison of weighted and unweighted generalized estimating equations (GEE) models.

Figure S1.

1. Chest – Mild melanosis

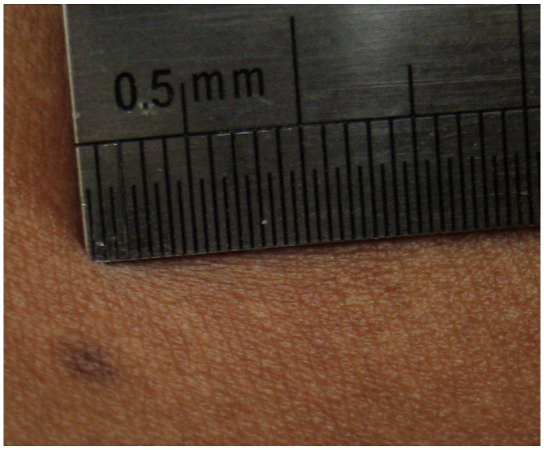

2. Chest -Severe melanosis

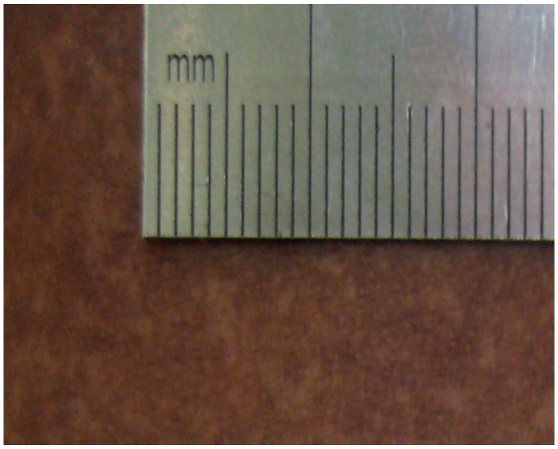

3.Palm – Mild keratosis

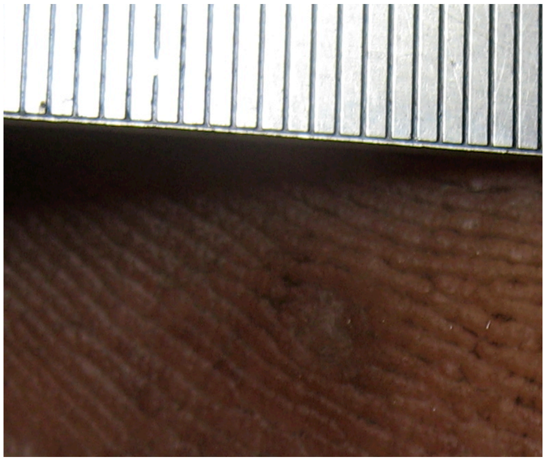

4.Palm - Severe hyperkeratosis

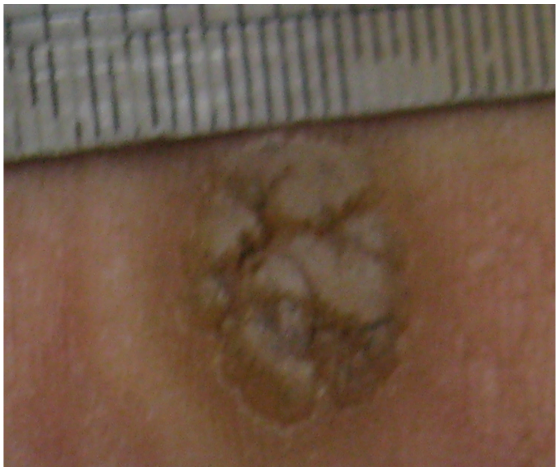

Colored photographs of skin lesions showing different types and levels of severity on patient's chest and palm

Table S1. Comparison of weighted and unweighted generalized estimating equations (GEE) models.

		Unweighted GEE				Weighted GEE [a]			
		Crude		Adjusted		Crude		Adjusted	
	Exposure (log10)	Mean Score Change	*p*-value	Mean Score Change	*p*-value	Mean Score Change	*p*-value	Mean Score Change	*p*-value
Decrease[c]	Water Arsenic	-0.84	0.27	-0.70	0.35	-0.90	0.23	-0.71	0.33
	Toenail Arsenic	-5.74	< 0.001	-5.22	0.003	-5.74	0.001	-5.17	0.003
Baseline[d]	Water Arsenic	-1.14	0.13	-1.34	0.08	-1.17	0.13	-1.39	0.07
	Toenail Arsenic	0.24	0.89	-0.092	0.96	0.26	0.88	-0.11	0.95

[a] Weights are estimated according to each individual's estimated probability of participating in the follow-up study using logistic regression on age, BMI and arsenic exposures at baseline.
